# *Ttc21b* Is Required in Bergmann Glia for Proper Granule Cell Radial Migration

**DOI:** 10.3390/jdb5040018

**Published:** 2017-12-19

**Authors:** Ashley M. Driver, Christopher Shumrick, Rolf W. Stottmann

**Affiliations:** 1 Division of Human Genetics, Department of Pediatrics, Cincinnati Children’s Hospital Medical Center, Cincinnati, OH 45229, USA; Ashley.Driver@uwsp.edu (A.M.D.); shumricm@mail.uc.edu (C.S.); 2 Division of Developmental Biology, Department of Pediatrics, Cincinnati Children’s Hospital Medical Center, Cincinnati, OH 45229, USA

**Keywords:** *Ttc21b*, cilia, *Shh*, cerebellum, postnatal, migration, Purkinje cell, Bergmann glia, Cre, LoxP

## Abstract

Proper cerebellar development is dependent on tightly regulated proliferation, migration, and differentiation events. Disruptions in any of these leads to a range of cerebellar phenotypes from ataxia to childhood tumors. Animal models have shown that proper regulation of *sonic hedgehog* (*Shh*) signaling is crucial for normal cerebellar architecture, and increased signaling leads to cerebellar tumor formation. Primary cilia are known to be required for the proper regulation of multiple developmental signaling pathways, including *Shh*. *Tetratricopeptide Repeat Domain 21B* (*Ttc21b*) is required for proper primary cilia form and function, and is primarily thought to restrict *Shh* signaling. Here we investigated a role for *Ttc21b* in cerebellar development. Surprisingly, *Ttc21b* ablation in Bergmann glia resulted in the accumulation of ectopic granule cells in the lower/posterior lobes of the cerebellum and a reduction in *Shh* signaling. *Ttc21b* ablation in just Purkinje cells resulted in a similar phenotype seen in fewer cells, but across the entire extent of the cerebellum. These results suggest that *Ttc21b* expression is required for Bergmann glia structure and signaling in the developing cerebellum, and in some contexts, augments rather than attenuates *Shh* signaling.

## 1. Introduction

Development of the cerebellum includes significant postnatal growth and morphological change. The most numerous cell type in the mammalian brain is the cerebellar granule cell, with estimates of approximately 100 billion cells in humans [1]. In mouse and human, cerebellar granule cells are produced postnatally in the external granule layer (EGL). The post-mitotic granule cells migrate radially on Bergmann glial fibers to the interior of the cerebellum and ultimately form the inner granule layer (IGL). In humans, disrupted cerebellar development can result in a wide variety of structural malformations, including cerebellar hypoplasia, failure of proper foliation, and tumor formation [2]. 

*Sonic hedgehog* (*Shh*) signaling has become a well-recognized pathway involved in early cerebellar development. Granule cell proliferation and proper development of the Bergmann glial migratory tracts are both known to require sonic hedgehog (SHH) secreted from Purkinje cells [3,4,5,6]. The downstream GLI transcription factors have dynamic expression patterns during early cerebellar development [7]. 

Altered levels of *Shh* signaling have severe cerebellar developmental consequences, and can cause tumor formation. Loss of *Shh* signaling results in a reduction of granule cell proliferation and cerebellar hypoplasia [8,9,10]. In addition to hypoplasia, conditional ablation of *Gli2* with an *En1-Cre* led to abnormal Purkinje cell and Bergmann glia morphology [8]. Deletion of *Gli1 and Gli2* leads to further disruptions in foliation and patterning [8]. In humans, impaired SHH signaling has been implicated in the cerebellar anomalies seen in both Joubert and Meckel Syndrome [11]. Conversely, activation of SHH signaling via loss of *Patched*-1 or constitutively activated Smoothened mutations results in medulloblastoma formation [12,13,14,15,16].

The primary cilium is a non-motile sensory organelle that is now well established to be critical for proper modulation of the SHH pathway [17]. Many proteins are trafficked through the primary cilium via intraflagellar transport (IFT). GLI proteins in particular require IFT through the primary cilium for proper processing into transcriptional repressor/activator forms [18,19]. Loss of cilia and/or IFT leads to multiple defects affecting SHH signal transduction, including perturbation of GLI processing patterns. Consistent with this role in SHH signal transduction, loss of primary cilia causes cerebellar hypoplasia [10] and modulates the effects of other mutations on tumor formation [14]. 

Among the IFT proteins, *Tetratricopeptide Repeat Domain 21B* (*Ttc21b*; *Ift139*) is required for normal ciliary retrograde transport [19] and early forebrain patterning and development [20,21]. Multiple studies have shown that the TTC21B protein (or the IFT139 orthologue) is found within the primary cilium [19,22,23]. Loss of *Ttc21b* has been shown to result in abnormal GLI3 processing, and thus increased *Shh* signaling [19,21]. In humans, *TTC21B* is one of the most commonly mutated genes in ciliopathy patients, and variants are present in disorders of the cerebellum such as Joubert Syndrome [22]. We hypothesized loss of *Ttc21b* during postnatal development may affect SHH signaling and cerebellar development. To investigate this and circumvent the perinatal lethality of *Ttc21b* mutants [19], we used the Cre-loxP system to separately ablate *Ttc21b* in Bergmann glia and Purkinje cells. Surprisingly, loss of *Ttc21b* resulted in ectopic granule cells in the posterior lobes of the cerebellum. Analysis of the affected lobes showed abnormal morphology of both Bergmann glia and Purkinje cells, as well as reduced SHH signaling. These results demonstrate a unique requirement for *Ttc21b* in cerebellar astrocytes and the first demonstration of reduced SHH signaling upon loss of *Ttc21b*. 

## 2. Materials and Methods 

### 2.1. Mouse Husbandry

All animals were maintained through a protocol approved by the Cincinnati Children’s Hospital Medical Center IACUC committee (IACUC2016-0098). Mice were housed in a vivarium with a 12-h light cycle with food and water ad libitum. All mouse alleles used in this study were previously published: *Ttc21b^alien^* is a null allele of *Ttc21b* [19], *Ttc21b^tm1a(KOMP)Wtsi-lacZ^* (*Ttc21b^lacZ^*) for analysis of *Ttc21b* expression via the LacZ expression cassette [24], *Ttc21b^flox^* [20,24]); a Cre recombinase reporter allele B6.129(Cg)-*Gt(ROSA)26Sor^tm4(ACTB-tdTomato^*^,*-EGFP)Luo*^/*J* (*ROSA^dTom^*^/*EGFP*^) [25]; FVB-Tg(GFAP-cre)^25Mes/J^ (GFAP-Cre) [26] and B6.129-Tg(Pcp2-cre)^2Mpin/J^ (Pcp2-Cre) [27]. *Gli1^tm2AlJ^*/*J* (*Gli1^lacZ^*) mice were used to measure *Gli1* expression [28]. For adult analysis, 4-month-old animals were sedated and transcardial perfusion with paraformaldehyde was performed using standard procedures. Brains were subsequently harvested and fixed for 48 h in 4% paraformaldehyde at room temperature (RT) and embedded in paraffin. For postnatal brain collection, pups were sedated with isoflurane to minimize discomfort followed by decapitation. Brains were collected and fixed for 24 h at 4 °C and embedded in O.C.T. medium (Tissue Tek) for cryosectioning or in paraffin. 

### 2.2. Histology and Immunohistochemistry

For histological analysis, brain sections were collected on a Leica microtome at a thickness of 5 μM. Sections were de-paraffinized, hematoxylin and eosin stained, and sealed using Cytoseal (Thermo Scientific, Waltham, MA, USA). Images were obtained with a Zeiss Stereoscope (all paired images are shown at the same magnification). For 3,3′-Diaminobenzidine (DAB) immunohistochemistry, sections were de-paraffinized and bleached with hydrogen peroxide. Sections were incubated overnight in primary antibody (NeuN, 1:500; Ki67 1:500) at RT. Sections were then washed in phosphate-buffered saline (PBS) and incubated for two hours in biotinylated swine-anti rabbit secondary antibody (1:500; Vector Laboratories, Burlingame, CA, USA) for 2 h at RT. Sections were then washed in PBS and incubated in avidin-biotin-complex (ABC) solution (Vector Laboratories) for 1 h at RT. Sections were then washed and visualized using DAB for 3 min followed by a PBS wash, ethanol dehydration, xylene incubation, and mounted with Cytoseal (Thermo). For fluorescent immunohistochemistry, sections were collected on a Leica cryostat at 12 μM thickness and washed in PBS prior to antigen retrieval with sodium citrate buffer (as appropriate for each antibody). Sections were then washed with PBS and incubated in blocking buffer (PBS + 4% normal goat serum and 0.1% Triton-X) for 1 h at RT. Sections were incubated overnight at 4 °C in primary antibody diluted in blocking buffer: Calbindin 1:1000 (Abcam, ab25085), glial fibrillary acidic protein (GFAP) 1:500 (Abcam, ab7260), and phospho-histone H3 (pHH3) 1:500 (SIGMA, H0412). Sections were then washed and incubated in Alexa Fluor-conjugated secondary antibodies diluted in blocking buffer (1:500, Thermo Scientific, Waltham, MA, USA) followed by PBS washes and DAPI counterstain (1:1000) for 10 min. To visualize the GFP expressed by the *ROSA^dTom^*^/*EGFP*^ Cre reporter allele, sections were only incubated for 1 h at RT with anti-GFP antibody (1:100, Life Technologies, A21311) followed by washes and DAPI counterstain. Sections were sealed with ProLong Gold (Life Technologies) and images were obtained on a Nikon C2 confocal microscope. All paired images were taken at the same magnification.

### 2.3. Section In Situ Hybridization and LacZ Staining

For RNA section in situ hybridization (SISH) and LacZ staining, sections were collected on a Leica cryostat at a thickness of 20 μM. The in situ hybridization was done according to standard protocols. For LacZ staining, sections were washed in PBS followed by fixation in cold 0.5% PFA on ice followed by PBS wash and incubation in LacZ buffer on ice. Sections were then incubated in X-Gal solution at 4 °C for 1–3 days, after which they were washed in PBS, counterstained in eosin, and dehydrated. Sections were sealed with Cytoseal (Thermo) and images were taken on a Zeiss Stereoscope with all paired images taken at the same magnification.

## 3. Results

### 3.1. Ttc21b Is Enriched in the Purkinje Cell Layer of the Developing Cerebellum and GFAP-Cre Ablation of Ttc21b Results in Ectopic Granule Cells in the Cerebellum

**Figure 1:**
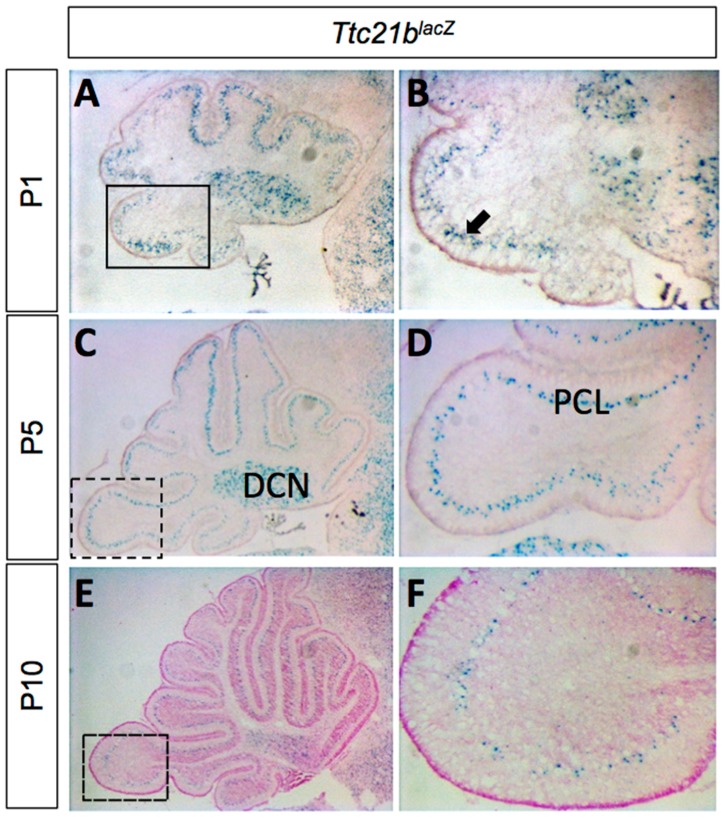
*Ttc21b* is expressed in the postnatal mouse cerebellum. (**A**–**F**) LacZ expression from the *Ttc21b^lacZ^* reporter is evident in the Purkinje cell layer of the cerebellum from P1–P10. Boxes in **A**,**C**,**E** indicate areas of focus in **B**,**D**,**F**, respectively. n ≥ 3 for LacZ staining. DCN: deep cerebellar nuclei; PCL: Purkinje cell layer.

We used a *Ttc21b^lacZ^* allele to determine the pattern of *Ttc21b* expression in the developing cerebellum. LacZ staining from P1–P10 showed expression in the Purkinje cell layer (PCL) and the deep cerebellar nuclei (DCN; Figure 1). The Purkinje cell layer at these stages is comprised of both Purkinje cells and the cell bodies of Bergmann glia [29]. 

Given the known roles of SHH signaling in early postnatal cerebellar development and the role of *Ttc21b* in regulating *Shh* signaling, we sought to determine if *Ttc21b* was required in the developing cerebellum. To circumvent the perinatal lethality of *Ttc21b* null mutations [19], we used a conditional genetic approach and performed a genetic ablation using GFAP-Cre [26] (Figure S1A–C). Consistent with previous reports, GFAP-Cre-stimulated recombination was observed in the telencephalic hemispheres and in the cerebellum. To confirm the cell types exhibiting Cre activity, we used a Cre recombinase reporter allele, *ROSA^dTom^*^/*EGFP*^, in combination with immunohistochemistry. We saw significant co-localization of GFP immunoreactivity indicating Cre activity with GLAST marking immature astrocytes (Figure S1G–I), but no such co-localization in the Calbindin-positive Purkinje cells (Figure S1D–F). 

**Figure 2:**
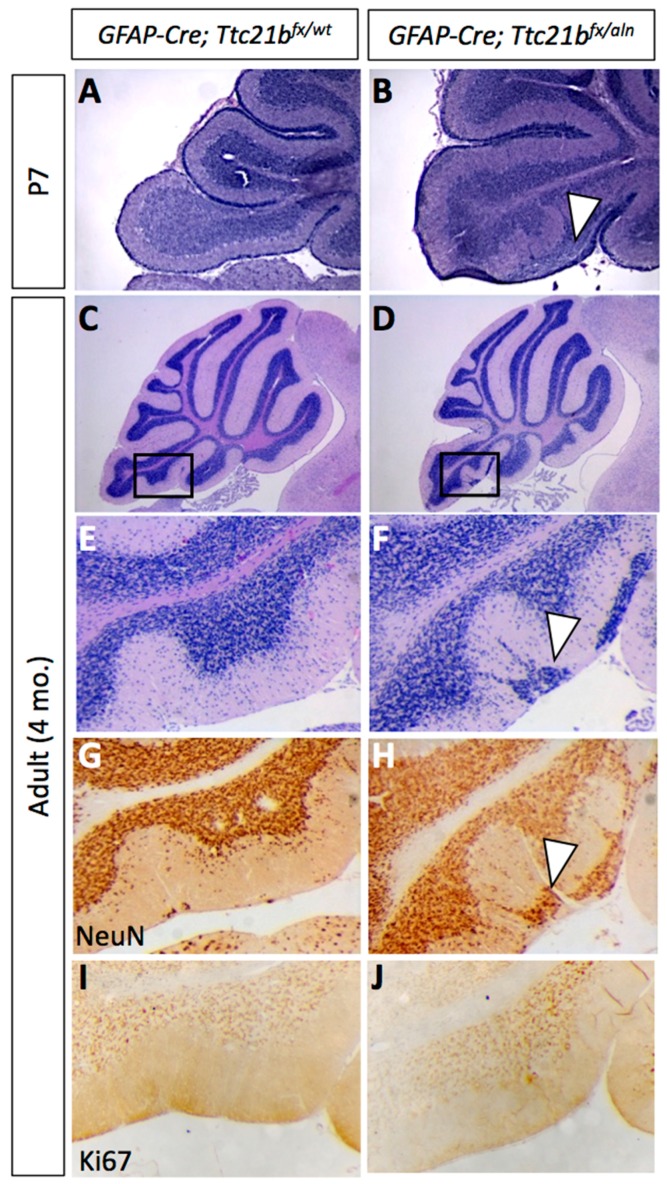
Ablation of *Ttc21b* from astrocytes results in ectopic granule cells in the cerebellum. H&E staining at P7 (**A**,**B**) and 4 months of age (**C**–**F**) show granule cells present in the outer layers of the cerebellum of *GFAP-Cre;Ttc21b^fx^*^/*aln*^ animals which are not found in controls (n > 4). NeuN immunoreactivity indicates that these ectopic cells are differentiated granule cells (**G**,**H**, arrowheads). Ki67 immunostaining for proliferative cells is negative in adults (**I**,**J**), suggesting that these are not proliferating tumors. n ≥ 4 for adult histology, n ≥ 3 for adult DAB staining and P7 histology.

We genetically ablated *Ttc21b* from Bergmann glia by crossing *GFAP-Cre;Ttc21b^aln^*^/*wt*^ mice with *Ttc21b^flox^*^/*flox*^ animals to generate *GFAP-Cre;Ttc21b^flox^*^/*aln*^ mice lacking functional *Ttc21b* in the entire GFAP-expressing lineage. Histological analysis of *GFAP-Cre;Ttc21b^flox^*^/*aln*^ animals at P7 showed dense areas of granule cells in the nodular region of the cerebellum which were not seen in control animals (Figure 2A,B, arrowhead; [30]. This phenotype was also observed in 4-month-old adult cerebella. Interestingly, the ectopic cells were found in the posterior cerebellum (lobes IX and X; Figure 2C–F). We demonstrated that these cells are differentiated granule cells with immunohistochemistry for NeuN as a marker for differentiated neurons. Ki67 immunostaining for proliferating cells indicated that these are not mitotically active (Figure 2G–J). This suggests that the ectopic cells, while displaced, are not forming tumors as has been previously shown upon manipulation of SHH signaling in the cerebellum.

### 3.2. Purkinje Cell and Bergmann Glial Cell Development Are Disrupted upon Loss of Ttc21b

We next sought to determine why the ectopic cells may be present in the *GFAP-Cre;Ttc21b^flox^*^/*aln*^ animals. We initially reasoned that they may be granule cells that did not properly migrate from the site of formation in the external granule layer. This migration occurs along the radially oriented Bergmann glial fibers as part of normal granule cell development [6]. To assess this hypothesis, we performed an immunohistochemical analysis of Bergmann glial cell development. 

**Figure 3:**
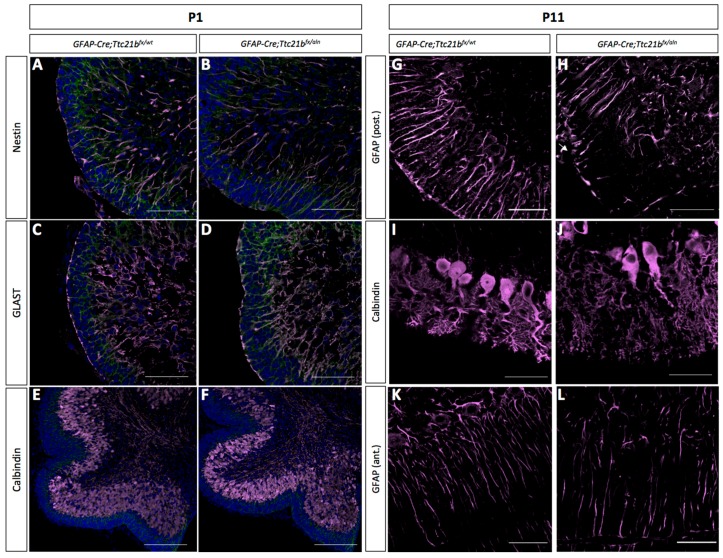
Purkinje cells and Bergmann glia development is disrupted in the *GFAP-Cre;Ttc21b^fx^*^/*aln*^ cerebella. (**A**–**D**) Immunostaining for immature astrocytes at P1 with Nestin (**A**,**B**) and GLAST (**C**,**D**) appears similar between control and *GFAP-Cre;Ttc21b^fx^*^/*aln*^ animals. (**E**,**F**) Calbindin immunostaining highlights the presence of Purkinje cells at P1 in both control and *GFAP-Cre;Ttc21b^fx^*^/*aln*^ animals. (**G**–**J**) P11 GFAP-positive mature astrocytes (**G**,**H**) and Calbindin-positive Purkinje cells (**I**–**L**) appear abnormal in the lower lobes of the *GFAP-Cre;Ttc21b^fx^*^/*aln*^ cerebella. (**K**,**L**) Anterior lobes still show normal Bergmann glia morphology in all animals. n = 3 for each genotype. Scale bars 50 μm.

Immature Bergmann glia were specified in approximately the appropriate numbers and appeared to have established radial fibers by P1, as shown by immunoreactivity for both Nestin and GLAST (Figure 3A–D). Purkinje cells were specified and migrated to the appropriate position in the *GFAP-Cre;Ttc21b^flox^*^/*aln*^ cerebella at P1 (Figure 3E,F). However, by P11 the *GFAP-Cre;Ttc21b^flox^*^/*aln*^ animals showed disrupted Bergmann glia fibers in the lower lobes of the cerebellum failing to form appropriate scaffolds for migration (Figure 3G,H). Interestingly, the anterior lobes where we did not note ectopic cells appeared to have normal Bergmann glia in the *GFAP-Cre;Ttc21b^flox^*^/*aln*^ animals (Figure 3K,L). At P11, the Purkinje cell bodies were irregularly aligned, and the dendritic arbors were more extensive in the *GFAP-Cre;Ttc21b^flox^*^/*aln*^ animals as compared to control (Figure 3K,L). 

### 3.3. SHH Signaling Is Reduced in GFAP-Cre;Ttc21b^flox/aln^ Cerebella

**Figure 4:**
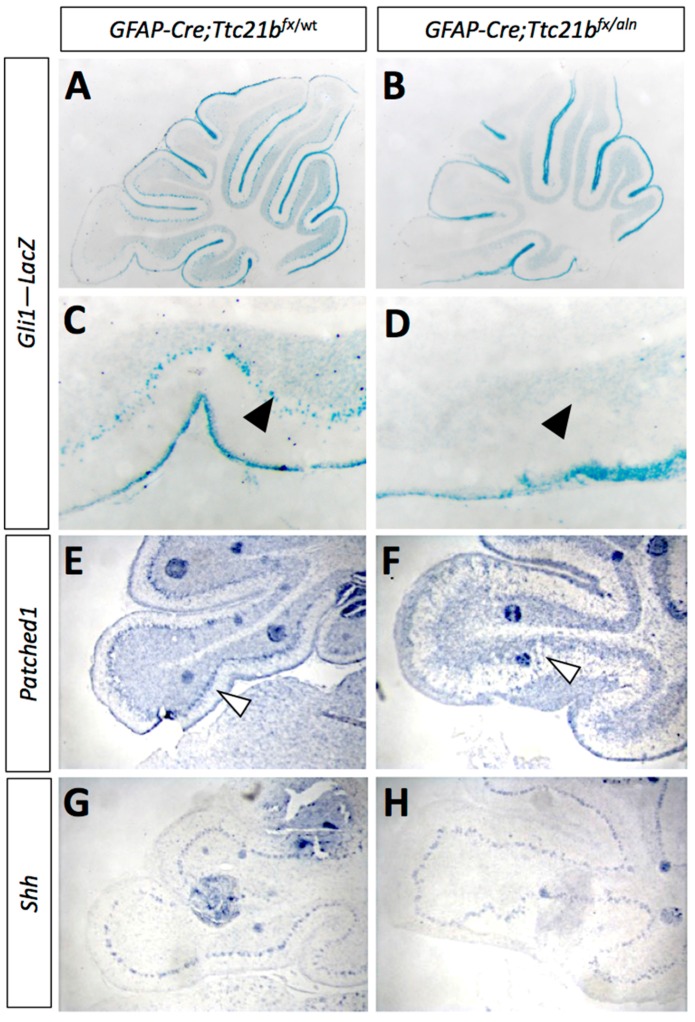
*Shh* signaling is reduced in *GFAP-Cre;Ttc21b^fx^*^/*aln*^ cerebella. (**A**–**D**) P11 *Gli1-*LacZ activity is reduced in in the Purkinje cell layer (arrows) of the *GFAP-Cre;Ttc21b^fx^*^/*aln*^ animals (**B**,**D**) as compared to control (**A**,**C**). (**E**,**F**) *Patched1* expression is also reduced in the *GFAP-Cre;Ttc21b^fx^*^/*aln*^ cerebella at P11 (**F**) compared to control (**E**), especially in the Purkinje cell layer (open arrowhead). (**G**,**H**) *Shh* expression appears similar between control (**G**) and *GFAP-Cre;Ttc21b^fx^*^/*aln*^ (**H**), but the organization of *Shh*-expressing cells again appears disorganized in the lower lobe of the cerebellum in ablated animals.

**Figure 5:**
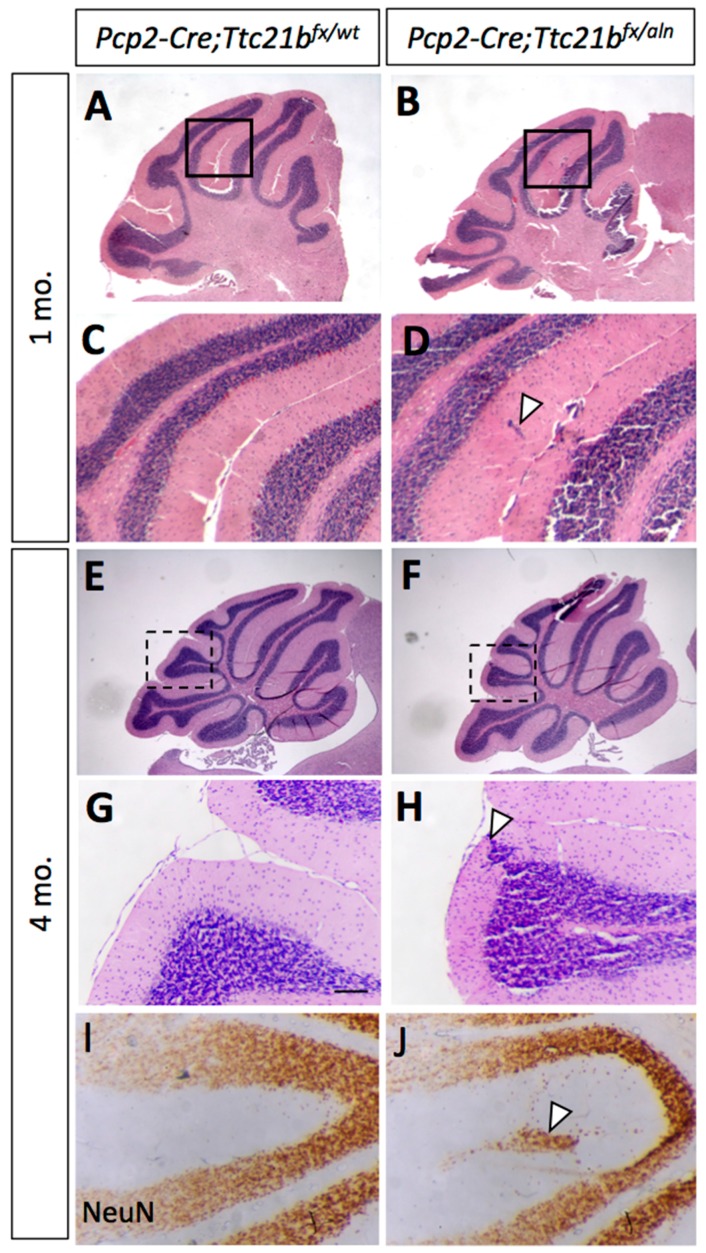
Ablation of *Ttc21b* with *Pcp2-Cre* results in similar, but milder, ectopic granule cell phenotype. (**A**,**B**) Small areas of displaced granule cells (arrowhead) are present in *Pcp2-Cre;Ttc21b^fx^*^/*aln*^ brains at 1 month of age. (**B**,**D**: boxes in **A**,**B** show areas highlighted in **C**,**D**). (**E**–**I**) These cells are still visible at 4 months of age in the *Pcp2-Cre;Ttc21b^fx^*^/*aln*^ animals (**H**, arrowhead) and are immuno-positive for NeuN, indicating that these cells are differentiated granule cells (**J**, arrowhead). n ≥ 3 for each genotype.

As the primary cilium is a major hub for *Shh* signaling and loss of *Ttc21b* has been shown to affect SHH signaling, we then investigated SHH pathway activity in the *GFAP-Cre;Ttc21b^flox^*^/*aln*^ animals with the *Gli1^lacZ^* allele [31]. *Gli1^lacZ^* expression was reduced in the Purkinje cell layer of *Gli1^lacZ^;GFAP-Cre;Ttc21b^flox^*^/*aln*^ animals (Figure 4A–D, arrow). Since *Gli1* is not expressed by Purkinje cells, these results suggest a loss of SHH signaling activity in Bergmann glia [7]. RNA section in situ hybridization for another *Shh* transcriptional target—*Patched-1* (*Ptch1*)—also showed reduced expression in the Purkinje cell layer of the *GFAP-Cre;Ttc21b^flox^*^/*alien*^ animals as compared to controls at P11 (Figure 4E,F, arrowhead). However, expression of the *Shh* itself was at similar levels between *GFAP-Cre;Ttc21b^flox^*^/*aln*^ and control animals, suggesting that Purkinje cell expression of the secreted SHH ligand is not affected (Figure 5G,H). Taken together, these data show that *Shh* signaling is reduced in the adult *GFAP-Cre;Ttc21b^flox^*^/*aln*^ cerebellum—a striking difference to previous observations of *Ttc21b* function in the nervous system. 

### 3.4. Purkinje Cell Ablation of Ttc21b Results in Milder Ectopic Granule Cell Phenotype

Since we observed *Ttc21b* expression in the Purkinje cell layers which have both Purkinje cells and Bergmann glia, we performed a similar genetic ablation of *Ttc21b* in the Purkinje cells with the *Pcp2-Cre* [27] (Figure S2). At 1 month of age, histological analysis again revealed small areas of ectopic cells in the outer layer of the *Pcp2-Cre;Ttc21b^flox^*^/*aln*^ cerebella (Figure 5A–D, arrowhead). This was also periodically seen in 4-month-old *Pcp2-Cre;Ttc21b^flox^*^/*aln*^ (Figure 5E–H, n = 4). Immunohistochemistry for NeuN again suggested that these cells were differentiated granule cells which failed to migrate to the IGL (Figure 5I,J). Interestingly, this phenotype was less penetrant than that in *GFAP-Cre;Ttc21b^flox^*^/*aln*^ animals, but was not solely restricted to the posterior lobes.

## 4. Discussion

Here we have demonstrated a role for *Ttc21b* in the both the Bergmann glia astrocytes and Purkinje cells of the developing mouse cerebellum. Using a *GFAP-Cre* genetic ablation of *Ttc21b*, we showed that a few granule cells are retained in the outer layer of the cerebellum, specifically in lobes IX and X. This appears to be a failure of granule cell migration, as the Bergmann glia—substrates for granule cell migration—have reduced radial projections in the lower lobes of the ablated animals. In contrast, a *Pcp2-Cre* Purkinje cell-specific ablation has a similar—but much less severe—phenotype, with only sparse areas of ectopic granule cells. Additionally, while GFAP-Cre recombination occurs in Bergmann glia, we noted effects on Purkinje cell development, supporting previous suggestions that a critical interaction between these two cells types is required for proper Purkinje cell dendritic development. We also observed that ablation of *Ttc21b* resulted in a reduction in SHH signaling in the Purkinje cell layer, which is the first time we have a reduction on the *Shh* pathway upon loss of *Ttc21b*. 

The radial fibers of the Bergmann glia serve as primary migratory tracts for granule cells in the developing cerebellum [6], and disruptions of these radial fibers have been previously shown to be associated with ectopic granule cells [32,33]. We only observed disruption of mature Bergmann glia in the affected lobes of the mutant, suggesting that this is a primary cause of the failed granule cell migration. Additionally, we noted abnormalities in Purkinje cell morphology in *GFAP-Cre;Ttc21b^flox^*^/*aln*^ animals consistent with numerous interactions between Bergmann glia and Purkinje neurons [34,35,36]. The ectopic granule cell phenotype was much less severe in the *Pcp2-Cre; Ttc21b^flox^*^/*aln*^ animals. This could suggest differing cell-specific requirements for *Ttc21b*. Indeed, patients with *TTC21B* variants have shown cytoskeletal abnormalities in human podocytes in addition to abnormal cilia, suggesting multiple roles for *TTC21B* [37]. Additionally, *Pcp2-Cre* recombination does occur later in development than GFAP-Cre (P5) and may not completely ablate *Ttc21b* until after many cells have already completed migration. 

Prior reports of *Ttc21b^aln^*^/*aln*^ mutants have shown increased SHH signaling in addition to abnormal GLI processing. Embryonically, loss of *Ttc21b* led to increased translation and abnormal proteolytic processing of GLI3 [19]. Inappropriate SHH activity has also been demonstrated in embryonic forebrain of *Ttc21b^aln^*^/*aln*^ mutants with abnormal GLI3 processing [19,21]. In a postnatal deletion of *Ttc21b*, increased GLI2 activity led to polycystic kidneys [24]. In contrast to these findings, we report a reduction in downstream *Patched* and *Gli1*, consistent with reduced *Shh* signaling. 

While *GFAP-Cre* should lead to loss of *Ttc21b* throughout the cerebellum, we only observed the ectopic granule cell phenotype in the posterior lobes. GLI2 is enriched in the Purkinje cell layer in the posterior lobes of the developing cerebellum around P5, and has been shown to be the major effector of positive SHH signaling [7]. While all three GLI transcription factors are expressed in the developing cerebellum, *Gli1* becomes restricted to the Bergmann glia in the Purkinje cell layer [7]. However, it is unlikely that the phenotype reported here is solely due to an effect on *Gli1*, as *Gli1* homozygous null mutants have a phenotypically normal cerebellum [38]. Both *Gli2* and *Gli3* null mice have abnormal cerebellar development, with primary defects in foliation [39]. Additionally, loss of *Gli2* has been shown to result in abnormal Bergmann glia morphology and Purkinje cell alignment [8]. Primary cilia are required for the proper processing of GLI2 protein into a transcriptional activator and GLI3 protein into a transcriptional repressor form. The loss of normal cilia function may have different effects on SHH signaling in different tissues, depending on which GLI protein has a more prominent role at that particular time in development. Given the enriched expression of *Gli2* in the same area as the ectopic cells and its role in stimulating SHH signaling, the reduced SHH noted here in the postnatal cerebellum may be more reflective of the loss of the GLI2 activator form than the GLI3 repressor.

The role of both ciliary genes and SHH signaling is a rapidly expanding field of study in human cerebellar development. SHH signaling is dysregulated in cells from Joubert syndrome and Meckel syndrome patients [11]. Interestingly, *TTC21B* variants are associated with Joubert syndrome patients [40]. Our study shows *Ttc21b* has a role in the glial cerebellar cells and loss of *Ttc21b* in different tissues can lead to increased and decreased SHH signaling.

## Supplementary Materials

The following are available online at www.mdpi.com/2221-3759/5/4/18/s1. Figure S1: GFAP-Cre recombination occurs in the Bergmann glia of the cerebellum, Figure S2: Pcp2-Cre recombination occurs in the Purkinje cells of the cerebellum.
