# Supplementary material for: Ttc21b Is Required in Bergmann Glia for Proper Granule Cell Radial Migration
Source: J Dev Biol. 2017 Dec 19;5(4):18. doi: 10.3390/jdb5040018 (PMC5831799; doi:10.3390/jdb5040018)
Supplement: Supplementary file 1 [file jdb-05-00018-s001.pdf]

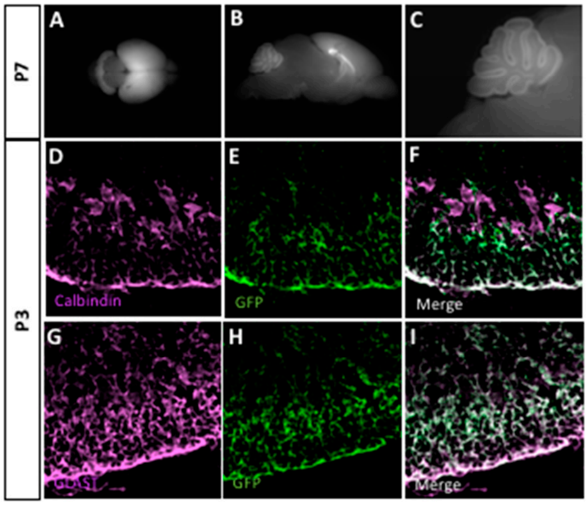

Figure S1: GFAP-Cre recombination occurs in the Bergmann glia of the cerebellum. (A,B) GFAP-Cre: $ROSA^{dTom/EGFP}$ GFP expression indicating Cre recombination appears in the cerebral cortex, hippocampus and cerebellum at P7. (D-H) Immunohistochemistry at P3 shows that the Purkinje cell marker Calbindin does not co-localize with GFP staining (marking Cre activity, D,G) while the astrocyte marker GLAST does strongly co-localize with GFP (E,H).

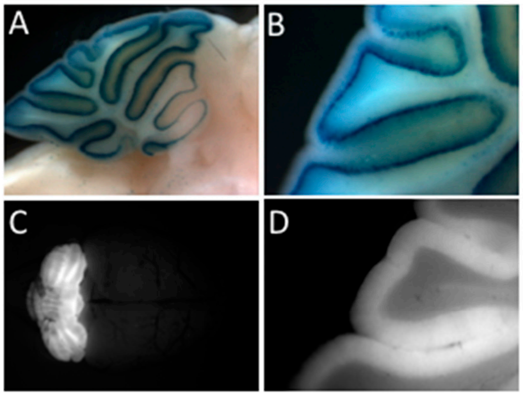

Figure S2: Pcp2-Cre recombination occurs in the Purkinje cells of the cerbellum. (A,B) Staining in a *Pcp2-Cre*;ROSA^{lacZ} cerebellum shows strong recombination in the Purkinje cell layer of the cerebellum at P10. (C,D) *Pcp2*-Cre: *ROSA*$^{dTom/EGFP}$ GFP fluorescence shows this signal is present in the cerebellum at 1 month of age.
